# The Impact of Poverty on Children’s Well-Being and Health Behavior Based on the Results of Research Conducted in One of Hungary’s Most Disadvantaged Micro-Regions

**DOI:** 10.3390/children11060624

**Published:** 2024-05-23

**Authors:** Gergely Fábián, Katalin Szoboszlai, Anikó Panna Tóth, Anita R. Fedor

**Affiliations:** Social Sciences Institute of Department of Social Sciences and Social Work, Faculty of Health Sciences, University of Debrecen, H-4400 Nyíregyháza, Hungary; fabian.gergely@etk.unideb.hu (G.F.); toth.panna@etk.unideb.hu (A.P.T.); fedor.anita@etk.unideb.hu (A.R.F.)

**Keywords:** child poverty, risk behavior, addictions

## Abstract

This empirical research on children’s poverty and the accompanying risk behavior was conducted in the Baktalórántháza micro-region, in one of the most disadvantaged micro-regions of Hungary. The study, completed in 2023, was conducted utilizing three methods, a questionnaire for families, interviews, and focus group interviews with social professionals working in the settlements. The region is one of the ten micro-regions with the highest poverty rate in the country. The majority of the population only has an elementary education, and the proportion of graduates is much lower than the national average. The proportion of households with three or more children is higher than the national average and the proportion of unemployed people in households with children is twice as high as the national average. Based on the experience of social workers working in the area, in addition to smoking and drinking alcohol, the consumption of psychoactive and psychotropic substances has increased among adolescents and young adults. Based on various indicators, children regularly consume illegal drugs. The origin and composition of these drugs are typically unknown. According to the reports by drug users, everyday life is easier, and they can escape from problems when under the influence of drugs. Based on the observations of experts, the consumption of various psychoactive substances has harmful effects on behavior, health, learning, and family life. School performance and the ability to think and learn decrease. Drug users are dissatisfied with their lives, have problems with social relationships, engage in partner violence, and may develop antisocial behavior in their lives.

## 1. Introduction

Research on child poverty began in Hungary twenty years ago. The next step was to develop and launch programs aimed at increasing children’s chances in the country’s poorest settlements [1,2,3,4]. Research based on quantitative methodology sought answers to the needs of households with children. In addition to the demographic data of the people living in the household, surveys conducted on a sample representative of the area concerned the characteristics of education, income, employment, housing, and children [5].

Poverty, in connection with the effects of inequality of life chances, means various disadvantages in the lives of children from the moment of their birth, reducing children’s opportunities for good education, work, income, and health as they are getting older [6]. If children cannot get access to the listed opportunities, the intergenerational transmission of poverty becomes permanent in adulthood [7].

This study presents a micro-settlement in eastern Hungary, mostly inhabited by poor people. Poverty and health-destroying drug use can harm the lives of children here. The settlements of the micro-region jointly applied for EU funding to increase children’s chances. The money was used for the development of child welfare services and the implementation of programs to help children’s socialization. In the settlements, the increase in employment and household incomes together with the improvement of the housing conditions led to the reduction of poverty; however, among the health-damaging behaviors, the spread of drug use has resulted in serious health and social problems, but these problems cannot be treated due to the lack of local services or specialists.

## 2. Materials and Methods

In the Baktalórántháza micro-region, child poverty and risk-taking behaviors have been studied several times. The researched micro-region is one of the most disadvantaged areas of the county, where poverty and the use of health-damaging substances endanger children, too.

The risk-taking behavior of young people living on the fringes of society was studied in 2017. A representative sample of 127 people in the Baktalórántháza micro-region was interviewed with the help of questionnaires, and focus group interviews were conducted with 22 people including social and health care professionals (N = 10), parents (N = 5), and young people (N = 7). The questionnaire and interview questions covered risk behaviors affecting children and young people, the consumption of health-damaging substances, and their possible solutions [8]. The young people and parents interviewed in the focus group interviews were mostly selected from the segregated area, and the experts were in daily contact with the residents of this part of town during their work. This research, which took place in May 2017, is the property of the Periphery Association, which implemented the research project KAB-KT-16-25568.

The most recent data collection was carried out in the Baktalórántháza micro-region in September 2023. It was conducted on a representative sample of families with children living in the micro-region. The methodology and the sample of the research was prepared and provided to the research group by the Child Opportunities Research Group of the Hungarian Academy of Sciences Center for Social Sciences. The data collection was performed using a mixed method. The data was collected in a total of 240 households using a questionnaire technique. We received the address list from the client of the research, in such a way that the sample is representative of the target group in the district. It was supplemented by a focus group interview as well as two individual interviews with social workers working in the micro-region.

Information from the sampled families was collected on the basis of a household questionnaire. The questions focused on the persons living in the household, the housing conditions, the available incomes, their sources, and the expenses typical of the household. In addition, a separate questionnaire focused on the children living in the household. This questionnaire inquired about the kindergarten and schooling characteristics, the children’s health status, the needs and demands, as well as the future vision related to the children [9].

The main goal of this study is to present the living conditions and characteristics of risk-taking behavior in households with children living in poverty in one of the most disadvantaged districts of the north-eastern region of Hungary, based on the 2017 and 2023 data collections.

The analytical work used the database of the two mentioned studies. The data compiled by survey data collection were analyzed by the SPSS 28 program package. Primarily descriptive statistics and cross-tabulation analysis were used. Interview data collection was also used in both studies. They were processed by using the method of content analysis. Furthermore, in addition to secondary analysis of statistical data related to the topic, professional strategic materials using the method of document analysis were also processed.

The two organizations—the Periphery Association in 2017 and the Child Opportunities Research Group of the Hungarian Academy of Sciences Center for Social Sciences in 2023—were our clients. We have obtained all the permissions to use the data and publish the research results. All data collection procedures were conducted ethically.

## 3. Results

### 3.1. Situation of the Baktalórántháza Micro-Region

The research was conducted in the north-eastern part of Hungary, in Szabolcs–Szatmár–Bereg county. This part of the country has various disadvantages compared to the central and north-western parts of the country. This county has the highest unemployment rate (first quarter of 2023: 9.3%), but the proportion of disadvantaged (2020: 35%) and cumulatively disadvantaged (2020: 23%) children is also the second highest among the 19 counties. The labor market situation is also greatly influenced by the educational composition of the population, which is also unfavorable in the county, as the proportion of those with only elementary education is significant [10]. The micro-region of Baktalórántháza is among the ten micro-regions with the highest poverty and deprivation rates in the country. The majority of the population (58%) in the micro-region have elementary education, and the proportion of the graduates is less than 6% [11].

Job opportunities are limited, and 10 of the 12 settlements are among those affected by significant unemployment [12]. The majority of the unemployed are undereducated and are over 45 years old [13]. As can be seen in Table 1 below, based on data from the TEIR databases, the district of Baktalórántháza is lagging far behind compared to the much more developed district of Nyíregyháza. For example, regular child protection support is shared by almost five times as many people based on their poor social situation, the number of registered job seekers is almost three times as many people, and it is typical that those who do work in the district are mostly employed in low-prestige employment groups. In 2011, approximately 45.4% of households in Baktalórántháza were unemployed. The proportion of apartments with a low level of comfort and occupied holiday homes in the district is also much higher than in the Nyíregyháza district. Another interesting fact is that the proportion of children in the district is higher and that of the elderly is lower than in the Nyíregyháza district, or even nationally, but of course the aging of the population is an observable trend here as well.

The county and within it the Baktalórántháza micro-region served as the location for analyses to prepare service planning. The Service Road Map (SZÚT) was completed in 2019 (and then updated in 2020). Its purpose was to study the type and extent of service gaps and which social group(s) is/are affected the most by these service gaps. In addition to the secondary analysis of the available documents (e.g., Local Equal Chance Program) and statistical databases (TEIR), the mapping of the service gaps was carried out through interviews, questionnaires, and the content analysis of the minutes of thematic meetings organized on special topics. One of the thematic sessions, entitled “*Possibilities of catching up for the disadvantaged children, compensation for disadvantage*”, took place in Baktalórántháza.

Based on the documents, the following problems emerged in the Baktalórántháza micro-region (Table 2). Ensuring access to early development is insufficient. The main reason for this is the lack of professionals. Primarily psychologists, speech therapists, teachers for children with special needs, developmental teachers, and kindergarten teachers would be needed. This was also highlighted by the respondents in the 2023 research. The lack of psychological, psychiatric, pediatric, and dental care was also mentioned among the shortcomings. A typical quote from our focus group interview with experts also shows this: “*Now I think that having a pediatrician here belongs to the category of completely unattainable dreams… The situation is that the medical care as well … There were three districts, we currently have two doctors, one retired and one non-retired. General practitioners … and then … the psychologist is really needed, this was replaced or helped by the Children’s Chance Program in the past periods. There is no psychologist anywhere in sight. We haven’t even mentioned the psychiatrists, but I think child psychiatry is a disaster at the county level. And nationally, it’s a disaster*” (Expert, Baktalorántháza, 2023).

Services ensuring quality free time for children are also incomplete, and the use of the Internet, which is now considered almost evident, has not been resolved among children living in extreme poverty. This can greatly limit the educational progress of the involved young people.

It would be important to organize various prevention programs for young people, for example on harmful addictions, drug consumption, healthy lifestyle, crime prevention, and family planning. Both the aforementioned documents and the specialists participating in the thematic session clearly pointed out that young people are not aware of healthy behavior or the effects of health-damaging activities. In addition, among the young people living in settlement-like conditions in the micro-region, the rate of early, unplanned childbearing is high compared to the national average.

The country has settlements and micro-regions with young age composition, where the proportion of the 0–14-year-old inhabitants is higher than the national average. The Baktalórántháza micro-region belongs to them. The aim is to assess and, if necessary, increase the number of nursery places in these settlements, as well as to establish equal opportunities for disadvantaged kindergarten and school children. Talent management and development programs are well justified in this social group. One of the basic criteria for creating opportunities for children living in the micro-region is the continuous contact with parents, because the disadvantages of children are also the disadvantages of their parents. The parents’ lack of knowledge affects the children and basically the whole family in a complex way [12,14,15].

In addition to the many disadvantages of the small region’s settlements, it should be mentioned that although the aging process of the population is also present here, its rate falls short of the national average. The proportion of people aged 65 and over remains below that of the age group of 14 and under.

The main results of the authors’ primary data collection/study conducted in the Baktalórántháza micro-region in 2023 are presented below.

#### 3.1.1. Demographic Situation

Based on the data of the 2022 census, the population of the micro-region is constantly decreasing compared to the 2011 census (as shown in Table 3), but at the same time, in most settlements of the micro-region the proportion of minors (0–14 years old) exceeds the national and Szabolcs–Szatmár–Bereg county ratios.

Although to a different extent, the decrease in the number of residents can be clearly measured in all settlements of the micro-region. The decrease in the number of the inhabitants is confirmed (among others) by an interview conducted in Ramocsaháza, where a social worker working in the area says: “*Well, our settlement is decreasing in population. Ten years ago, there were 1572 people, the death rate is also very high, the birth rate can be said to be low. There are years when there are many pregnant mothers, and there are years when there are very few*”.

Table 4 shows the proportion of children aged 0–14 in the district based on data from the 2022 Census. Based on the data, it can be established that with the exception of Nyírkércs and Petneháza, the rates exceed the data typical for the county and the country (16.6% and 14.7%, respectively). It is also confirmed by the result that an average household includes four persons in the sample, compared to the national average of 2.36 persons. However, the decrease can also be easily measured in this respect, since in 2011, during another survey that was also carried out in the Baktalórántháza micro-region, the average household size was 5.21 persons. The number of children has also decreased in the last decade, since in 2011 the average number of 0–17-year-old children living in households was 2.3, while in 2023 it was 1.81.

In 2023, an average of 2.23 children were born among the surveyed families, including children who no longer live in the given household. Only three people indicated that one of their children was temporarily or permanently in state care.

#### 3.1.2. Characteristics of Housing

The floor area of the apartments/houses in the micro-region is 104 square meters on average (60, 80, 90, 100, and 110 square meters are the most common ones), but houses of 20 and 300 square meters can also be found. According to legal title, the vast majority (93%) live in their houses as owners, 1.7% rent the apartments from the municipality, 0.8% from a private person, while 4.6% live in the property with another legal title (e.g., they can use the apartment as a favor).

Each apartment has a kitchen, and they consist of several rooms. Ten percent of the apartments do not have bathrooms, and 12% of the apartments do not have flush toilets. The vast majority of the apartments/houses have mains water supply (94.2%), 5% typically do not have mains water, and 0.8% have domestic water supply. The typical problems concerning the apartments and the neighborhood are summarized in Table 5. According to the respondents, the most frequently mentioned problems in the neighborhood were the lack of garbage collection in the street (16.7%) and crime, vandalism, and violence (15.5%). Bad housing conditions, e.g., damp, moldy walls, contribute to the development of various health problems and respiratory diseases. It is typical for 88% of the houses to have gardens suitable for cultivation. Sixty-two percent grow vegetables, 60% grow fruit in their gardens, but only 30% keep farm animals.

According to 13.3% of the respondents, alcohol consumption is very typical in their living environment, and 10.4% said drug consumption was (also) very typical.

#### 3.1.3. Employment

Half of the respondents have jobs and work as employees. The second most common category, 17.2%, includes those who use various subsidies for childcare (GYES, GYET, GYED, CSET). GYES = child care allowance, GYET = childrearing support, GYED = childcare benefit, and CSET = baby care aliment. The public sector employed 9.6%, casual work was typical for 3.3%, while 3.8% received regular income as individual entrepreneurs (or working in their own business). The proportion of the unemployed is 4.6% overall, where 2.5% receive benefits while 2.1% are without benefits. A typical quote from our focus group interview captures the employment situation and challenges well: “*…those who actually want to work have to commute, so they have to commute to Nyírbátor or Nyíregyháza, and not many families with small children can afford these, where one parent works, it would be difficult for the other parent to go to three shifts, so someone has to stay at home to take care of the children in Hungarian, where there are small children. And there is no work around here. The only thing that provides employment in the area is the local government within the framework of public works*” (Expert, Petneháza, 2023).

The vast majority of the employed work either at their place of residence or in a nearby settlement. Their employment is low-status and minimum-income jobs, like communal work at the municipality and trained work required for food processing.

These are typically indicators of precariousness, with precarious characteristics predicting social relegation, with the realistic alternative of becoming precarious [17].

#### 3.1.4. Income Situation

The net average wage measured in the micro-region shows a lag compared to the national data, as it was 371,800 HUF in July 2023 (385,600 HUF if discounts are taken into account). The average monthly net income per capita in the micro-region is 89,175 HUF, which is lower than the monthly net income measured in the Northern Great Plain region and in Hungary. If the average income measured in the micro-region is taken into consideration, it should be emphasized that 52% of the respondents have an income below the average.

Studying the composition of incomes, regular wages and salaries from full-time jobs are dominant, which are typical for 151 households (average value: 325,168 HUF), and since families with children live in households, the family allowance (210 households; average value: 26,357 HUF) should also be taken into consideration.

#### 3.1.5. The Situation of Families with Children and Their Expectations for the Future

In the micro-region the proportion of households with three or more children is higher than the national average. In households with children the unemployment rate is almost twice as high as the national average. Under-education is also typical, where the proportion of people who completed a maximum of eight classes of primary school is high [18]. One third of children are brought up in families without active earners, and more than one quarter of poor families live exclusively on social transfer income [19].

It is interesting that in spite of this fact parents are optimistic about their children’s future, and their expectations regarding their children’s future education are high [18]. In the research conducted by the authors in 2023, it was also found that although the respondents saw the present and the near future as gloomy, despite their difficulties, they were particularly optimistic about the futures of their children. The majority of parents (94%) talk to their children about their future. In this regard, the parents expressed optimism, nearly half of them believe that the children will obtain at least secondary education, and a similar proportion believes that the children will become professionals with a degree. In light of this it is not surprising that in several dimensions they believe that their children will have a more favorable life situation at the age of 20 than their parents have currently.

### 3.2. Characteristics of Drug Use and Risk-Taking Behavior

Among the behaviors harmful to children’s health, those related to drugs prove to be worrisome in the child population living in poverty. The definition of drug is interpreted comprehensively, including all natural or artificial substances that affect the central nervous system and change its functioning when they enter the human body. Based on this, for example, nicotine, caffeine, alcohol, medicines, and psychoactive substances are classified as drugs. In the poor culture, the consumption of nicotine, alcohol, and cheap drugs made at home from easily available, fast-acting, and constantly changing chemical substances has also reached children.

In Hungary poverty and drug use are one of the research areas of social work. Szécsi and Sik studied drug use in segregated settlements of a disadvantaged micro-region and the results confirm the general use of smoking in all age groups, starting from adolescence, to regular alcohol consumption common among the parents’ generation, and drug addiction, which primarily means the use of sedative drugs. In addition to classic drugs, new psychoactive drugs that cause rapid stupor have recently appeared in the drug use of people living in segregated communities.

Typically, children try psychoactive drugs during prepuberty and puberty, because they do not know their health-damaging and life-threatening effects. The explanation of drug use is that the use of new psychoactive drugs are antidotes to boredom, and they help to forget about poverty in the settlements [20]. Further studies exploring the substance use habits of marginalized social groups show that deprived social groups, like the poor living in segregated areas, are especially exposed to the danger of the consumption of extremely cheap, new psychoactive drugs.

In these groups the consumption of new psychoactive drugs significantly exceeds the national average [21]. According to Rácz and his colleagues, “*when formulating possible responses to the addiction of people living in extreme poverty, it is very important to take into account the perspective of people who face the difficulties and hopelessness of their everyday lives where substance use can be functional and can be a part of their survival strategy*” [21].

The research of Lannert and his colleagues on the drug use of elementary school students living in segregated schools presents the background and types of drug use: “Alcohol, smoking cigarettes or weed help these children escape from stress or from a bleak environment with little stimulation, it’s a shocking experience that they even “consume” rat poison or screw loosener, just to get intoxicated” [22]. Drug use is the symptom, where children use mind-altering drugs to escape from reality.

Children will be easy targets to try and consume drugs if there are drugs in the children’s families, in school, and in their living environment. The new psychoactive drugs, designer drugs, are easily available for them, but the composition of these drugs is often unknown, and their consumption is harmful to health and even life-threatening. Prepubertal and pubertal drug users have a high chance of developing drug addiction and have a significant risk of leaving school early, early sexual intercourse, or crime [22].

Children living in the settlements of the Baktalórántháza micro-region, especially children who are more likely to be affected by risk behavior and who live in segregated areas, were included in the research conducted in 2017 in order to explore the risk behavior and drug use habits of children living on the social periphery. Among the studied adolescents, in addition to poverty, there were also risk behaviors that harm children’s health and affect their future [8].

This research covered the drug-related activity of children and young adults. Based on the answers, smoking and alcohol consumption were more typical of young age groups than drug consumption. Seventy percent of young people smoked and 46.5% smoked on a daily basis. Alcohol consumption was typical for 62%, the frequency of alcohol consumption varied, and in many cases it lasted until drunkenness. The influence of friends and schoolmates can be seen in individual consumption habits. Twenty-five people in the sample mentioned that they are drug users; they most often use marijuana, synthetic cannabinoids, sleeping pills, and other sedative drugs. Among the new psychoactive drugs, home-made compounds can be found. Their combined chemical effects are not precisely known; thus, these drugs cause serious health damage or death. As a local social worker said in our focus group interview, it is a complex problem: “*So it is very difficult to find a job, one part of my clients works in public works, the other part is unemployed at home. I think quite a few of them, the parents, are alcoholics. Almost all of the clients’ children also smoke, the teenagers with whom I have a slightly more confidential relationship already regularly consume alcohol, I know that they also use some other substance. I think the situation of this area is very desperate*”.

A significant difference in the drug consumption of boys and girls was shown for 76 children between the ages of 12 and 18 in the Baktalórántháza region. Substance use was higher among boys than among girls. Drug use is more common among young people over the age of 18 than among those under 18, but very young children in the district also try drugs; this is illustrated by the quote below, which was told by a young person from Baktalorántháza who was not even 12 years old at the time during our focus group interview: “*First, there was a B… Gabi in the children’s home. He told us to go smoking weed. I went with him, I inhaled it, I started to feel dizzy, I couldn’t see, I fell, I couldn’t see where I was going*”. At the same time, there is no significant difference in terms of the types of school that the young people attend, or what kind of education their parents have, or where they live (independently or together with parents, or possibly with foster parents, etc.).

Half of the respondents consume tobacco products, and one third of them smoke an average of five cigarettes per day. Regarding alcohol consumption, almost half of the respondents do not drink alcohol. The majority of consumers drink alcohol once a month or less often, and while half of the respondents do not get drunk from drinking, one third of them occasionally get drunk, and every twelfth respondent has gotten drunk from alcohol more than ten times. Children and young people consume the following drugs, although not with regular daily use: herbal, new psychoactive substances, over-the-counter sleeping pills, sedatives, marijuana, organic solvents, and alcohol with medication.

The results of the authors are similar to Csák et al.’s (2020) results from research on the use of psychoactive substances. According to them, most often, new psychoactive substances and not classic drugs are used by people living in rural segregated areas. “*The majority of high-risk drug users living in segregated environments smoke regularly and drink alcohol very often, but a quarter of them regularly, almost every day use sleeping pills or sedatives without medical advice. … The occurrence of both synthetic cannabinoids and synthetic cathinones was significant, but the frequency of trying “classic” drugs was lower than them. It basically shows a different pattern compared to the entire population*” [21].

Based on the experience of the Baktalórántháza Regional Social Center—Family and Child Welfare Service, in addition to smoking and alcohol consumption, the consumption of psychoactive and psychotropic substances is spreading significantly among local adolescents and young adults. Based on the signals received by the service, 35 young adults and those living in their surroundings, including children, regularly consume illegal drugs and psychoactive substances.

The origin and the composition of the drugs are usually unknown among consumers, which poses a huge danger to young people. According to the reports of drug users, everyday life seems easier under the influence of drugs, problems fade into the background, and they can run away from problems. Based on the observations of local specialists, the consumption of various psychoactive substances has harmful effects on behavior, health, learning, and family life in the short and long term. The behavior of drug users is extremely strange and unpredictable, they are full of anxiety and fear, they hallucinate, and they cannot distinguish between real and unreal things. As a local expert said in our focus group interview in 2017: “*Young people living in residential homes use these drugs in even greater numbers. This is based on proven facts, so the problem here is that no one among consumers is really aware of what kind of health damage these so-called adulterated or non-adulterated drugs can cause in the life of a child or family, so there are absolutely none, in fact not yet we don’t know either. … we used to say that we are happier with a person who is “only” an alcoholic, because these drug users are unpredictable*”.

Furthermore, the risk of heart attack or stroke increases. School performance decreases and the risk of dropping out increases. The ability to think and learn decreases. The drug users will not be satisfied with their lives, and social relationship problems, partner violence, antisocial behavior, theft, lying, and financial difficulties may develop in their lives. There is a greater chance that they will not have jobs or income, and poverty and the lack of a future will continue in their lives [19,23].

Poverty is not only a marginal situation for children and young people in terms of deprivation, but risk behaviors like drug use, prostitution, and crime are also more common in their environment. Prostitution and sexual exploitation are a big problem for the studied settlement. Women and girls sell their bodies voluntarily or under coercion, but the money received for sex work is not always theirs.

Young girls and women are at high risk of becoming victims of sexual exploitation, violence, and human trafficking. Cases of crime are related to violations of rules and drug use. Possession and distribution of drugs is a crime. Distributors easily involve children in crimes because they may receive a lighter sentence due to their age. The presence of prostitution and crime in the family environment is a source of danger for children and young people to follow family patterns and health-damaging behaviors.

### 3.3. Experiencing Health and Social Crisis Situations

In the case of pregnant mothers in a social crisis, local social experts and the Health Visitor Service try to ensure that children to be born are brought up in their families with professional assistance and complex family care.

In terms of health risks, the number of cases of termination of unwanted pregnancies among children is increasing, even though health visitors organize regular health, prevention, and educational presentations for elementary school and high school students. In previous years the Family and Child Welfare Service and the Family and Children’s Welfare Center provided intrauterine devices free of charge to the applicants to prevent “unwanted pregnancy” [23].

Another area where local specialists are experiencing an increase is the number of mental illnesses in the child and adult population. The already poor mental state of the population was further worsened by the COVID-19 pandemic, where many people lost their loved ones, and it was more difficult to access health services. In addition, due to the proximity of the border, the threat of the war in Ukraine and existential uncertainty increased. Prices increased, many people in the micro-region were faced with the fact that they could not even buy basic food, and the number of people in fee arrears increased.

Family helpers report an increase in the frequency of domestic violence, the number of suicide attempts among young people, and the consumption of alcohol and psychoactive substances. Elementary school teachers report aggression, abuse, and fights. Insults and fights often escalate to the point where not only is school equipment damaged, but they also endanger children’s physical health [23,24]. In Hungary, during the COVID-19 pandemic closures in the spring of 2020, every fifth student could not participate in online education (based on research by the MTA TKI—Supported Research Group of the Hungarian Academy of Sciences, UPR report submitted by members of the Child Rights NGO Coalition (Hungary) 2021). The transition to online education was especially difficult for disadvantaged families, the necessary tools were not available, and internet connection and digital illiteracy were also serious obstacles [24]. The digital competence level of not only children and parents but also teachers is low (UPR report submitted by members of the Child Rights NGO Coalition (Hungary) 2021). The situation confirmed that there is a great need for the development of digital literacy in the training and further training of teachers and social professionals.

In 2022, compared to the previous year, the number of registered children and families in risk shows an increase. The cases that have arisen are increasingly complex and complicated, and they often show serious danger [23]. “*The problems are built on each other, and they are interrelated. One problem generates another. Long-term unemployment causes financial problems, which can lead to living difficulties, housing problems, family conflicts, lifestyle problems, mental and health problems, and the development of addictions. The child, as the symptom bearer, reacts to problems within the family with behavioral disturbances and deviant actions. These processes result in the development of child-rearing problems*” [19].

Child neglect, child-rearing problems, bad school behavior, and achievement disorders are common in the micro-region. Their reason is usually that “Parents from low-income families are confronted with multiple stressors and challenges, such as financial stress, work-related stress (working multiple jobs, poor working conditions and non-standard work schedules), and coping with their physical and mental health problems with lack of or limited access to health care services. All of these stressors may contribute to poor parenting practices” [25].

Families have special importance because they basically determine the children’s physical and mental development, the transmission of social values, and the development and strengthening of personality traits. A well operating family can protect children against anxiety due to disappointments, it can strengthen self-confidence, and through education it helps young people to be open, cooperative, and tolerant. Family means mental protection, which protects them from the use of illegal substances, harmful addictions, and from getting to the social periphery [26].

## 4. Discussion

The purpose of the research was to present the living conditions and characteristics of risk behavior in households with children living in poverty in one of the most disadvantaged districts of the north-eastern region of Hungary. The results of the authors’ primary data collection conducted in 2023 and the data of the previous questionnaire research in 2017 were analyzed. Additionally, interviews with local experts were conducted, and a secondary analysis of statistical data and analysis of professional strategic documents were carried out.

The results were divided into three large groups. First, the situation of the micro-region of Baktalórántháza was introduced. The demographic, housing, employment, and income situation was presented, focusing on families with children and their future expectations. Next, the characteristics of drug use and risk-taking behavior were presented, which were followed by the experience of health and social crisis situations.

Taking the situation in the micro-region into consideration, it can be said that the present results are consistent with the results of other research investigating the micro-region of Baktalórántháza [8,10,11,12,14,15,18,19]. According to the results of our research, the micro-region is struggling with extremely complex problems. Poverty is high, the education level of the population is low, the unemployment rate is extremely high, and the proportion of children with cumulative disadvantages is high. The population’s access to services is inadequate, especially the access to early development.

Based on our results, it can be said that there is a large shortage of professionals in the micro-region, there are not enough psychologists, speech therapists, special education teachers, developmental teachers, kindergarten teachers, and even pediatric and dental care is not properly resolved. There is a great demand for services that would help children spend their free time actively and compensate for school disadvantages, creating equal opportunities, like the Tanoda program. This program has been operating in many places in the country. It is necessary to increase the number of nursery school places in the micro-region, and there is also a need for talent development programs to support disadvantaged children. According to experts, it is a big problem that young people have children too early and they do not plan to have children either. Prevention, information, family planning, and the possibility of contraception should be supported. Prevention is also important in relation to substance abuse and crimes since they are also dominant problems in the micro-region.

In relation to the demographic situation, although the proportion of minors in the micro-region is higher than the national or even the county average, it can be said that overall, the decline of the resident population is a typical trend here as well.

Housing conditions are bad, ten percent of the properties are without bathrooms, twelve percent of the apartments do not have flush toilets, and five percent typically do not even have running water. Damp, moldy walls contribute to the development of various health problems and respiratory diseases. Poor housing conditions have a negative impact on the population’s health and children’s life chances.

Half of the respondents were employed and had workplaces. The most employed people either work in their place of residence or in a nearby settlement, typically in low-status and minimum-income jobs, for example as community workers or semi-skilled workers. The net average wage measured in the micro-region lags behind the national data.

One third of children are brought up in families without active earners at all, and more than one quarter of poor families live exclusively on social transfer income [19]. It was very interesting that despite their poor situation, most parents with young children were very optimistic about their children’s futures, they believe that they will obtain secondary or even higher education, and that they will be in a much more favorable life situation than their parents.

The results of a previous study carried out in the micro-region [18] also showed that parents’ expectations regarding their children’s future education are high. It is something that can be “built on”, where parents naturally want their children to have better life, and they see learning as important and try to support their children in it despite the difficulties. Parents can therefore be cooperative and motivated despite their difficult situation, but of course an appropriate level of education, concrete help, and opportunities are needed so that the children can achieve the desired higher education, so that they can break out of poverty.

Also, according to our research results the occurrence of risky behaviors such as drug use, prostitution, and crime are also common in the micro-region. They are all very challenging problems and are closely related to poverty.

In addition to smoking and drinking alcohol, the consumption of psychoactive and psychotropic substances is spreading significantly among local adolescents and young adults. The origin and composition of the drugs are usually unknown among consumers and pose a danger to young people. According to the reports of drug users, everyday life seems easier under the influence of drugs, problems are pushed into the background, and they can escape from problems—this is the purpose and motivation of consumption. However, by using drugs, they become trapped, and as a result, their situation typically worsens, and poverty and futurelessness continue in their lives [19,23].

Local specialists see an increase in the number of mental illnesses, both in the child and adult population. The already poor mental state of the population was further worsened by the COVID-19 pandemic and the war situation in Ukraine. Existential uncertainty increased, prices increased, and many people in the micro-region were faced with the fact that they could not buy even basic food. The number of people in arrears increased.

The frequency of domestic violence and the number of suicide attempts among young people, the consumption of alcohol and psychoactive substances, the number of aggressions, the amount of abuse, and the number of fights between children in primary school have increased [23,24]. The number of registered children and families at risk is increasing, severe child neglect and child-rearing problems are common, and cases are becoming more and more complex and complicated, showing high risk [23].

Specialists and services are needed to prevent children from being at risk and to manage problems. “*Early childhood care in Hungary has an exceptionally good and diverse network, even in international comparison: there are paediatricians, health visitors, nurseries, kindergartens, which in principle could mean an opportunity to help all children, including disadvantaged and cumulatively disadvantaged children and their families, as well as to compensate for these disadvantages. However, for a variety of reasons, these services are provided little or not at all for the most disadvantaged families and children*” [19].

One of the reasons, for example, is that services are difficult to reach in these disadvantaged areas, where they struggle with a large shortage of professionals, and additionally, most families do not have the chance to use the various health services, private crèches, kindergartens, and developments on a market basis [27,28].

The results of the research of the authors confirm this. It is a particularly big and worrying problem that children in disadvantaged areas like the Baktalórántháza micro-region have the least access to early development opportunities. According to the results of the authors’ research, there is a large shortage of professionals in the micro-region of Baktalórántháza. There is a need for pediatricians, psychologists, speech therapists, teachers for children with special needs, developmental teachers, and kindergarten teachers. Due to poor financial and housing conditions, children are more likely to get sick, and it is also typical that they receive diagnosis and treatment late, which also affects their future life and future chances. Greater attention should be paid to prevention and early development [18].

Poverty, low income, and low socioeconomic status are also related to behavioral problems in children and adolescents [25].

It would be important for decision-makers to realize that child poverty has very high costs in the long term, increases health and social expenses, and reduces productivity; this is why it is in everyone’s common interest to invest in children and reduce child poverty [29].

As the WHO emphasizes, “one of the most significant factors hindering the improvement of population health and the eradication of health-related inequalities is childhood poverty and the transmission of poverty between generations” [30].

If the goal is to improve the health of the population, the most important thing is to fight against child poverty. However, it is not enough to only deal with children—complex help is needed. This includes supporting families, improving the relationship between parents and children, and involving parents in school programs. There would be a great need for the development of parenting skills, psychological assistance, management consulting, and the organization of parenting courses. Of course, it is not enough in itself, and unemployment in parents, financial difficulties of families, and inadequate housing and hygiene conditions are social problems that must be addressed in order to better the well-being of families and children [23].

According to the local specialists, “*In order for children’s chances to improve in the region, their direct support is not enough. It is also important to indirectly help parents in managing their failures and their life, as well as improving the quality of their living environment. In parallel with the correction of the currently existing problems, it is also important to start preventative activities in the areas of health preservation, family planning, career guidance, the development of addictions, and life management and child-rearing counselling. Persistent poverty and exclusion exclude certain members of society from access to needs and services that ensure well-being. It further determines the current situation of disadvantaged children, the happy experience of childhood, and, on the other hand, through unequal access to educational, cultural, sports, social, health, etc. services and through insufficient social relations—it essentially limits their adult opportunities*” [19].

In connection with drug use, it would of course be important to have locally available services that can manage drug use problems; however, such interventions alone are not enough, and it would be important to improve the situation of those living in segregated areas, with appropriate social policy and labor market measures to reduce the poverty and hopeless situation of those living here [21].

There is a need to continue the locally available successful programs (health prevention presentations, group work, parent training, catch-up and playhouse activities, school crime prevention, strengthening of the child protection signaling system, etc.), although their funding is currently uncertain. Due to the exposure to tender opportunities, local professionals find it difficult to plan in advance.

## Figures and Tables

**Table 1:** Some features of the Baktalórántháza micro-region.

	Baktalórántháza Micro-Region		Nyíregyháza Micro-Region
	2011	2022	2022
Number of settlements belonging to the micro-region:	14	12	15
Number of inhabitants	19,629 persons	17,609 persons	162,229
Number of inhabitants over 65 (the number of people per permanent resident aged 0–14)	68.6	94.8	126
Proportion of 65–x-year-old people (percentage)	12.2	15.4	18.7
Proportion of 0–14-year-old people (percentage)	17.7	16.2	14.9
Average monthly number of people receiving regular child protection support (number per 100 0–18-year-olds)	n.a.	39.1 (2022)	8.1
Number of Internet subscriptions on xDSL network per 1000 inhabitants (pc)	n.a.	41.4 (2022)	54.1
Number of residents per GP and pediatrician GP (persons)	1784.5	2201.1 (2022)	1590.5
Available hospital beds per 1000 inhabitants (pcs)	0	0	15.9
Percentage of the population belonging to the Gypsy (Romani, Beas) nationality (percentage)	9.5	n.a.	3.1 (2011)
Proportion of people employed in high-prestige employment groups (percentage)	8.7	12.2 (2022)	28.1
Registered jobseeker, (15–64 age group, person)	16.3	8.3	2.5
Proportion of unemployed households (percentage)	45.4	n.a.	n.a.
Proportion of apartments with low level of comfort and occupied holiday homes (percentage)	19.3	n.a.	7.1 (2011)

Source: TEIR 2011, 2022 [13].

**Table 2:** Summary table of service gaps identified in the micro-region.

**Equal chances for children**	- programs promoting healthy nutrition and a healthy lifestyle - early development - nursery care - the possibility of practicing outdoor forms of exercise - taking care of children during the holidays - specialist pediatric care on site - programs promoting staying in the settlement - available leisure and sports activities during the summer holidays
**Equal chances for people living in deep poverty and the Romas**	- labor market opportunities - debt management - uniform information service on available services - digital competence development - help with family planning
**Equal chances for women**	- alternative daytime (and occasional) childcare - access to social and leisure programs (baby–mother club) - legal aid available in case of domestic violence or other harm - locally available health screenings - availability of atypical forms of employment
**Equal chances for people with disabilities**	- accessibility of information communication - the possibility of using health and screening tests - access to labor market services - access to work
**Equal chances for the elderly**	- access to specialist medical services and screening programs - programs to help prevent victimization - dementia mitigation support service - the possibility of acquiring necessary IT skills - residential care for the elderly - conditions for exercising

Source: Service Road Map of Szabolcs–Szatmár–Bereg county, 2020 [14].

**Table 3:** Change in the number of residents in the micro-region.

Settlement	Population (Person)	Change Compared to 2011 (%)
Baktalórántháza	3434	−12.3
Berkesz	778	−10.0
Nyírjákó	821	−5.6
Nyírkarász	2225	−4.1
Nyírkércs	700	−11.8
Nyírtass	1791	−11.2
Ófehértó	2412	−4.1
Petneháza	1533	−14.2
Ramocsaháza	1368	−9.8

Source: KSH, Census, 2022 [16].

**Table 4:** The proportion of children aged 0–14 in the micro-region (%).

Settlement	Proportion of Children Aged 0–14 (%)
Baktalórántháza	19.3
Berkesz	18.6
Nyírjákó	18.6
Nyírkarász	15.1
Nyírkércs	11.0
Nyírtass	15.9
Ófehértó	15.7
Petneháza	14.5
Ramocsaháza	17.0

Source: KSH, Census, 2022 [16].

**Table 5:** Problems characteristic to the apartments and the neighborhood (%).

There is no garbage collection in the street	16.7
Crime, violence, vandalism in the area	15.5
The floor and walls are wet	10.5
The window frame and the floor are rotting	9.2
There is not enough light, the apartment is too dark	9.2
The roof is leaking	7.5
The neighbors are noisy or there is a lot of noise from the street	7.5

Source: Own data collection, 2023.

## Data Availability

The data presented in this study are available on request from the corresponding authors. The data are not publicly available due to privacy.
